# Macronutrient Intake and Risk of Crohn’s Disease: Systematic Review and Dose–Response Meta-Analysis of Epidemiological Studies

**DOI:** 10.3390/nu9050500

**Published:** 2017-05-15

**Authors:** Lirong Zeng, Sheng Hu, Pengfei Chen, Wenbin Wei, Yuanzhong Tan

**Affiliations:** Department of Gastroenterology, The Central Hospital of Enshi Autonomous Prefecture, Enshi 445000, China; hushenwhu@163.com (S.H.); chenpengfeienshi@sohu.com (P.C.); tanyuanzhongenshi@sohu.com (Y.T.)

**Keywords:** macronutrient intake, Crohn’s disease, disease risk, dose–response, meta-analysis

## Abstract

Dietary intake is potentially associated with the onset of Crohn’s disease (CD), but evidence from epidemiological studies has remained unclear. This study aimed to evaluate the role of macronutrient intake in the development of CD. A systematic search was conducted in PubMed and Web of Science to identify all relevant studies, and the role of macronutrients in the development of CD was quantitatively assessed by dose–response meta-analysis. Four case-control studies (a total of 311 CD cases and 660 controls) and five prospective cohort studies (238,887 participants and 482 cases) were identified. The pooled relative risks (RR) for per 10 g increment/day were 0.991 (95% confidence interval (CI): 0.978–1.004) for total carbohydrate intake, 1.018 (95% CI: 0.969–1.069) for total fat intake, and 1.029 (95% CI: 0.955–1.109) for total protein intake. Fiber intake was inversely associated with CD risk (RR for per 10 g increment/day: 0.853, 95% CI: 0.762–0.955), but the association was influenced by study design and smoking adjustment. In subtypes, sucrose intake was positively related with CD risk (RR for per 10 g increment/day: 1.088, 95% CI: 1.020–1.160). Non-linear dose–response association was also found between fiber and sucrose intake and CD risk. In conclusion, this meta-analysis suggested a lack of association between total carbohydrate, fat or protein intake and the risk of CD, while high fiber intake might decrease the risk. In subtypes, high sucrose intake might increase the risk of CD.

## 1. Introduction

Crohn’s disease (CD) is an intestinal inflammatory disorder of unknown etiology, and its incidence has been steadily on the rise [1,2]. The disease is clinically characterized by abdominal pain, diarrhea and high rates of surgery caused by intestinal stenosis and perforation. The main therapeutic strategy is to alleviate active inflammation, maintain remission and reduce the rates of relapse and surgery. Non-steroidal anti-inflammatory drugs (e.g., 5-aminosalicylic acid (5-ASA)) are effective for mild to moderate disease, while corticosteroids, immunosuppressors (e.g., azathioprine), or biological agents (e.g., infliximab) are required for moderate to severe disease or when 5-ASA proves ineffective [3,4]. However, drug dependence, side effects, medical costs and poor life quality impose a heavy burden on CD patients. Thus, it is necessary to identify the etiology or risk factors, and prevent the disease from the source. Diet is usually considered to a potential pathogenic factor, especially for westernized dietary habits [5,6]. Secondly, the treatment with elemental diet and food exclusion has a similar effect to the drug therapy [7,8]. As a result, it was believed that certain food or nutrients might play an important role in the pathogenesis of CD. For dietary food, high consumption of fruit was reported in inverse association with the risk of CD (odds ratio (OR): 0.57, 95% confidence interval (CI): 0.44–0.74), but not in vegetables (OR: 0.66, 95% CI: 0.40–1.09) [9]. For macronutrients, fat, carbohydrate, fiber or protein intake was not significantly associated with another type of inflammatory bowel disease, ulcerative colitis [10,11]. However, the role of macronutrient intake is controversial in the pathogenesis of CD, and no meta-analyses have concentrated on this. Therefore, we conducted a systematic review and dose–response meta-analysis to quantitatively assess the role of macronutrient intake in the development of CD.

## 2. Materials and Methods

### 2.1. Search Strategy

The databases of PubMed and Web of Science were searched for relevant studies published up to 16 March 2017, using the key words including “diet*”, “carbohydrate”, “sugar”, “fiber”, “fat*” and “protein” in combination with “inflammatory bowel disease” or “Crohn’s disease”. Studies in languages other than English or Chinese were excluded. Moreover, we also reviewed the references of related studies and reviews for undetected studies.

### 2.2. Study Selection and Exclusion

Two authors (L.Z. and P.C.) reviewed the studies independently. The inclusion criteria were as follows: (i) case-control or cohort-based study design; (ii) contained at least three quantitative categories of macronutrient intake; (iii) evaluated the association between macronutrient intake and CD risk; (iv) presented relative risk (RR), OR, or hazard ratio (HR) estimates with 95% CI. The exclusion criteria were as follows: abstracts without full texts, reviews, case reports and pediatric studies.

### 2.3. Data Extraction and Quality Assessment

Two authors (L.Z. and P.C.) extracted the data by a standardized collection form. All differences were resolved by discussion with a third author (W.W.). In each study, the following information was extracted: first author, publication year, area, study design, follow-up year in prospective-designed studies, diagnostic criteria, number of cases/controls, age distribution, time at diagnosis and retrospective period in case-control studies, exposure categories, effect sizes (RR, HR, OR) with 95% CI and adjusted factors. The Newcastle–Ottawa Scale (NOS) was used to assess the methodological quality of included studies [12].

### 2.4. Statistical Analysis

For the low incidence of CD, OR and HR were roughly regarded as the RR in this study [10,11,13]. The assigned dose in each category was defined as the mean intake. If the mean intake per category was unavailable, we chose the midpoint of the upper and lower boundaries in each category as the assigned dose. For open-ended lower and upper categories, we defined the lowest boundary as zero and the open-ended interval length as the same with the adjacent category respectively. Groups were regarded in equal size or follow-up when cohort size or person-year per category was unavailable, and the case number per category was obtained by the method of Bekkering et al. [14]. In the Reif et al. study [15], the missing 95% CIs were obtained according to the method of Orsini [16]. In the Jantchou et al. study [17], we converted the unit of grams per kilogram of body weight to grams per day by multiplying an average weight of 60 kilograms. In the Chan et al. study [18], the person-years were calculated assuming that the interval between recruitment and diagnosis was the follow-up period.

As the cut-off points for categories varied among the studies, the RR for per 10 increment of exposure in each study was estimated before pooling the risk estimates between studies using a random-effect model by the method of Greenland and Longnecker [19] and Orsini et al. [20]. Moreover, a potential non-linear dose–response association between nutrient intake and CD risk was modeled by using restricted cubic splines with three knots at percentiles 10%, 50% and 90% [21]. The Wald test was chosen to evaluate linear or non-linear trends [22].

Subgroup analyses were conducted on study design, cohort and smoking adjustment to evaluate the stability of main results The Egger’s test was used to detect publication bias [23]. The heterogeneity among studies was estimated by *Q* test and *I*^2^ statistic [24], and *I*^2^ > 50% represented substantial heterogeneity. All statistical analyses were performed with Stata SE12.0 software (StataCorp LP, College Station, TX, USA).

## 3. Results

### 3.1. Study Characteristics

The search strategy resulted in 12,155 records: 10,980 from PubMed, 1119 from Web of Science and 16 through other sources. After excluding duplicated and irrelevant records, eight records (nine studies) were included in this meta-analysis [15,17,18,25,26,27,28,29] (Table 1). Persson et al. [25] classified the results by sex, which were divided into two separate reports. The two studies by Ananthakrishnan et al. [27,28] were based on the same cohort, and focused on fat and fiber respectively. Among these eight studies, four were case-control designed with a total of 311 CD cases and 660 controls, while the other five were prospective cohort designed with a total of 238,887 participants and 482 cases. Validated semi-quantitative food frequency questionnaires (FFQ) contained various food items and consumption frequency, and were used to measure macronutrient consumption in all studies. The results in most studies were statistically adjusted for certain factors, such as age and energy intake. In case-control studies, dietary habits before diagnosis were obtained to guarantee the pre-illness dietary intake. In quality assessment, the included studies had an average score of 7.78.

### 3.2. Carbohydrate Intake and CD Risk

**Figure 1:**
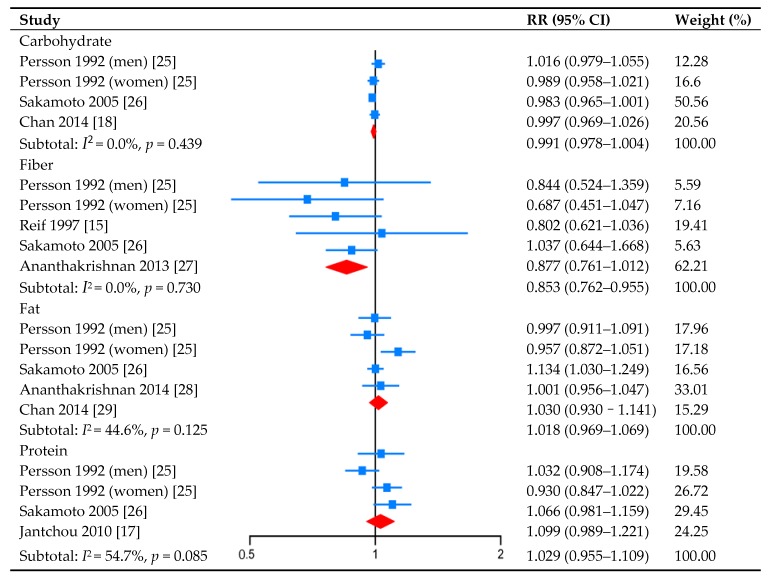
Forest plots (random-effect model) of meta-analyses on the association between carbohydrate, fiber, fat and protein intake (per 10 g increment/day) and the risk of Crohn’s disease.

Four studies investigated the association between carbohydrate intake and CD risk (Figure 1). No studies showed a significant association, and no evidence of a non-linear relationship between them was detected (*p* for non-linearity was 0.376). The pooled RR was 0.991 (95% CI: 0.978–1.004, *I*^2^ = 0.0%, *p_heterogeneity_* = 0.439) for per 10 g increment/day in carbohydrate intake.

### 3.3. Fiber Intake and CD Risk

**Figure 2:**
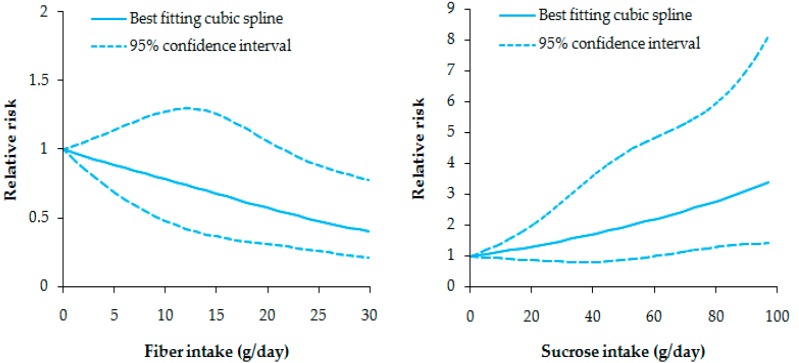
Non-linear dose–response analysis of fiber and sucrose intake and the risk of Crohn’s disease.

Five studies reported the association between fiber intake and CD risk (Figure 1). The pooled RR was 0.853 (95% CI: 0.762–0.955, *I*^2^ = 0.0%, *p_heterogeneity_* = 0.730) for per 10 g increment/day in fiber intake, suggesting a protective role in the development of CD. We also found a non-linear relationship between them (*p* for non-linearity was 0.019) (Figure 2).

### 3.4. Fat Intake and CD Risk

Five studies investigated the association between fat intake and CD risk (Figure 1). Only the study of Sakamoto et al. showed a significant association (RR: 1.134, 95% CI: 1.030–1.249), and no evidence of a non-linear relationship between them was detected (*p* for non-linearity was 0.281). The pooled RR was 1.018 (95% CI: 0.969–1.069, *I*^2^ = 44.6%, *p_heterogeneity_* = 0.125) for per 10 g increment/day in fat intake.

### 3.5. Protein Intake and CD Risk

Five studies reported the association between protein intake and CD risk (Figure 1). No studies showed a significant association, and no evidence of a non-linear relationship between them was detected (*p* for non-linearity was 0.163). The pooled RR was 1.029 (95% CI: 0.955–1.109, *I*^2^ = 54.7%, *p_heterogeneity_* = 0.085) for per 10 g increment/day in protein intake.

### 3.6. Intake of the Nutrients’ Subtypes and CD Risk

Four studies reported an association between the intake of sugar subtypes and CD risk, including monosaccharide, disaccharide, starch and related subtypes (Table 2). Only sucrose was found to be significantly related with CD risk, and the pooled RR was 1.088 (95% CI: 1.020–1.160, *I*^2^ = 0.0%, *p_heterogeneity_* = 0.395) for per 10 g increment/day. A non-linear relationship was also found with a *p* value of 0.023 for non-linearity (Figure 2). Three studies reported an association between intake of fat subtypes and CD risk, including saturated fatty acid (SFA), monounsaturated fatty acid (MUFA), polyunsaturated fatty acid (PUFA) and their subtypes. No fat subtypes showed a significant association with CD risk. In the Jantchou et al. study [17], animal-or vegetable-derived protein showed no significant association with CD risk (the highest vs. the lowest category:RR: 2.700, 95% CI: 0.690–10.520; RR: 1.040, 95% CI: 0.280–3.800).

### 3.7. Subgroup Analysis and Publication Bias

Subgroup analyses were conducted on study design, cohort and smoking adjustment. No substantial changes of primary results were found between groups except for fiber intake (Table 3). The protective role of fiber intake in the development of CD was weakened by the risk factor of smoking (RR for per 10 g increment/day: 0.890, 95% CI: 0.776–1.020). The Egger’s test detected no obvious publication bias in fiber (*p* = 0.708), while there were not enough studies to conduct the analyses for carbohydrate, fat and protein.

## 4. Discussion

To the best of our knowledge, this is the first dose–response meta-analysis to study the role of macronutrient intake in the development of CD. Our results suggested a lack of association between dietary carbohydrate, fat or protein intake and CD risk, which was inconsistent with the conventional views. The Western lifestyle is characterized by high consumption of carbohydrates, fats and protein, and the prevalence of westernized dietary habits in Asian cohorts coincides with an increasing incidence of CD in those regions. Thus, these macronutrients were usually thought to be risk factors in the development of CD. Cola drinks and chocolate are rich in carbohydrates and fats, and in the study of Russel et al., both were found in positive association with the development of CD (OR (95% CI): 2.2 (1.5–3.1); 2.5 (1.8–3.5)) [30]. However, in the Racine et al. study, the dietary pattern of “high sugar and soft drinks” showed no significant association with CD risk (the highest vs. the lowest category: RR (95% CI): 1.48 (0.60–3.61)), as well as the pattern “animal fats, seafood, potatoes and alcohol” (RR (95% CI): 0.71 (0.29–1.73)) [31]. It was controversial on the role of these macronutrients in the development of CD, and environmental and genetic factors might contribute to the inconsistency in findings between studies [32,33].

For carbohydrate intake, we detected no significant association with CD risk. However, as another major subtype of carbohydrate, high fiber intake could decrease CD risk. The result was consistent with the meta-analysis of Liu et al., which also found dietary fiber intake could reduce the risk of CD (the highest vs. the lowest category: RR (95% CI): 0.44 (0.29–0.69)) [34]. In subgroup analysis, when not adjusted for smoking, a lower RR was observed with higher fiber intake, but the same finding was not present with an adjustment for smoking. This indicated that a low fiber might be simply associated with smoking (which is a risk factor for Crohn’s disease), rather than being an independent risk factor for CD [35]. Furthermore, the limited number of studies might also contribute to the inconsistency. Third, the protective role of fiber might be weakened by the risk factor of smoking.

As another major subtype of carbohydrate, sugar, was not associated with CD risk in this study, which was consistent with the study of Racine et al. However, in respect to sugar subtypes, high sucrose intake was positively associated with CD risk. In animal models, high consumption of dietary sucrose could induce the inflammation of multiple tissues [36,37,38]. Secondly, high intake of dietary sucrose could trigger endoplasmic reticulum (ER) stress which was also associated with the pathogenesis of Crohn’s disease [39,40]. Wang et al. also reported a positive association between sucrose intake and the risk of ulcerative colitis (RR for per 10 g increment/day: 1.098, 95% CI: 1.024–1.177). Thus, we thought that high intake of dietary sucrose could increase the risk of CD [11].

This meta-analysis study has several strengths. First, to our knowledge, this is the first dose–response meta-analysis to identify the role of macronutrients in the development of CD. Second, only studies with at least three quantitative categories of exposure were included which demonstrated a higher quality than those with two categories, and somewhat covered the limits in the number of studies [41]. Third, subgroup and sensitivity analyses were used to test the stability and reliability of primary results, and the results were consistent in general. There were also a few limitations in this study. First, the inclusion of case-control studies might introduce certain bias, such as recall bias, which might potentially lead to differential misclassification of various types of exposure, and exaggerate or weaken the effect estimates. Second, not all potential confounders were adjusted in each study.

## 5. Conclusions

This meta-analysis suggested a lack of association between total carbohydrate, fat or protein intake and the risk of CD. High fiber intake might decrease the risk of CD, but the association was influenced by study design and smoking adjustment. In subtypes, high sucrose intake might increase the risk of CD. Large-scale prospective designed studies are needed to confirm our findings.

## Figures and Tables

**Table 1:** Characteristics of included studies.

First Author, Year, Area	Study Design	Diagnostic Criteria	Cases/Controls (Age)	Time at Diagnosis (Retrospective Period ^#^)	Exposure Categories (Dietary Assessment)	Risk Estimates (95% CI)	Adjusted Factors
Persson, 1992, Sweden (for men) [25]	Population-based case-control	The scoring table suggested by Lennard-Jones	63/147 (15–79 years)	Within 4 years (5 years ago)	T3 vs. T1	Relative risk	Age, energy intake
					Protein	2.2 (0.7–6.9)	
					Carbohydrate	2.1 (0.5–8.1)	
					Fat	1.3 (0.4–4.4)	
					Fiber	1.2 (0.5–2.6)	
					(Validated FFQ)	-	
Persson, 1992, Sweden (for women) [25]	Population-based case-control	The scoring table suggested by Lennard-Jones	89/158 (15–79 years)	Within 4 years (5 years ago)	T3 vs. T1	Relative risk	Age, energy intake
					Protein	0.4 (0.2–1.3)	
					Carbohydrate	1.0 (0.2–4.3)	
					Fat	0.7 (0.2–2.9)	
					Fiber	0.4 (0.2–1.0)	
					(Validated FFQ)	-	
Reif, 1997, Israel [15]	Population/clinic-based case-control	-	33/144 (mean, 29.12/29.45 years)	Within 1 year from onset of symptoms (before the illness and symptoms began)	T3 vs. T1	Odds ratio	Age, sex, country of origin, residential neighborhood, energy intake
					Fiber	0.40 (0.10–1.65)	
					(Validated FFQ)	-	
Sakamoto, 2005, Japan [26]	Hospital-based case-control	The criteria of the Research Committee on Inflammatory bowel disease in Japan	126/211 (15–34 years)	Within the past 3 years (5 years before the time of the study)	Q4 vs. Q1	Odds ratio	Age, sex, study area, education, smoking habits
					Protein	2.06 (0.99–4.28)	
					Fat	2.86 (1.39–5.90)	
					Carbohydrate	0.53 (0.27–1.03)	
					Fiber	0.90 (0.43–1.86)	
					(Validated FFQ)	-	
Jantchou, 2010, France (for women) [17]	Prospective cohort study	Clinical, radiological, endoscopic and histological criteria	30/67, 504 (mean, 50.9/52.8 years)	Within a median of 54.5 months (a mean follow up of 10.4 years)	T3 vs. T1	Hazard ratio	Body weight, energy intake
					Protein	3.34 (0.90–12.4)	
					Carbohydrate	1.31 (0.42–4.14)	
					Fat	0.98 (0.25–3.88)	
					(Validated FFQ)	-	
Ananthakrishnan, 2013, USA (for female registered nurses) [27]	Prospective cohort study	Typical symptoms ≥ 4 weeks; endoscopy; histology; radiography	269/170, 169 (NHS I: 30–55 years; NHS II: 25–42 years)	With a median age of 54 years at diagnosis (NHS I from 1984 to 2006; NHS II from 1991 to 2007)	Q5 vs. Q1	Hazard ratio	Age, cohort, smoking, BMI, oral contraceptive use, use of post menopausal hormone therapy, regular use of NSAIDs, regular use of aspirin, energy intake
					Fiber	0.59 (0.39–0.90)	
					(Validated FFQ)	-	
Ananthakrishnan, 2014, USA (for female registered nurses) [28]	Prospective cohort study	Typical symptoms ≥ 4 weeks; endoscopy; histology; radiography	269/170, 169 (NHS I: 30–55 years; NHS II: 25–42 years)	With a median age of 54 years at diagnosis (NHS I from 1884 to 2006; NHS II from 1991 to 2007)	Q5 vs. Q1	Hazard ratio	Age, cohort, smoking, BMI, oral contraceptive use, use of post menopausal hormone therapy, regular use of NSAIDs, regular use of aspirin, energy intake
					Fat	0.98 (0.66–1.45)	
					(Validated FFQ)	-	
Chan, 2014, Europe [18]	Prospective cohort study	Radiology; endoscopy; histology	110/440 (50.1 years/50.1 years)	More than 18 months after recruitment (from 1991–1998 to 2004–2010)	Q5 vs. Q1	Odds ratio	Age, sex, center, recruitment date, follow-up period, energy intake, BMI, metabolic rate, physical activity, smoking
					Carbohydrate	0.87 (0.24–3.12)	
					(Validated FFQ)	-	
Chan, 2014, Europe [29]	Prospective cohort study	Follow-up questionnaire, in-patient record, histology database, medical note	73/292 (50.5 years/50.2 years)	More than 18 months after recruitment (from 1991–1998 to 2004)	Q5 vs. Q1	Odds ratio	Age, sex, center, recruitment date, smoking, total energy intake, BMI, dietary vitamin D and relevant fatty acids
					Fat	1.42 (0.26–7.67)	
					(Validated FFQ)	-	

^#^ Retrospective period in case-control studies, and follow-up period in prospective cohort studies; T, tertile; Q, quartile; BMI, body mass index; FFQ, food frequency questionnaire.

**Table 2:** Intake of the nutrients’ subtypes (per 10 g increment/day) and the risk of Crohn’s disease.

Subtypes	Included Studies	RR (95% CI)	*I*^2^ (%)
Sugar	Reif 1997 [15]; Chan 2014 [18]	0.998 (0.969–1.027)	0.0
Monosaccharide	Persson 1992 (men) [25]; Persson 1992 (women) [25]	0.971 (0.715–1.317)	49.9
Fructose	Reif 1997 [15]	0.843 (0.695–1.023)	-
Disaccharide	Persson 1992 (men) [25]; Persson 1992 (women) [25]	0.988 (0.871–1.121)	0.0
Sucrose	Persson 1992 (men) [25]; Persson 1992 (women) [25]; Reif 1997 [15]	1.088 (1.020–1.160)	0.0
Starch	Chan 2014 [18]	0.994 (0.946–1.044)	-
Fat			
SFA	Sakamoto 2005 [26]; Ananthakrishnan 2014 [28]	0.980 (0.843–1.140)	17.2
MUFA	Sakamoto 2005 [26]; Ananthakrishnan 2014 [28]	1.137 (0.842–1.536)	78.8
Oleic acid	Ananthakrishnan 2014 [28]; Chan 2014 [29]	1.015 (0.900–1.144)	0.0
PUFA	Sakamoto 2005 [26]; Ananthakrishnan 2014 [28]	1.306 (0.816–2.092)	76.2
Arachidonic acid	Ananthakrishnan 2014 [28]	0.000 (0.000–721.226)	-
Linoleic acid	Ananthakrishnan 2014 [28]; Chan 2014 [29]	1.097 (0.871–1.383)	0.0
α–linoleic acid	Chan 2014 [29]	0.035 (0.000–3.299)	-
DHA	Chan 2014 [29]	0.004 (0.000–1706.027) ^#^	-
EPA	Chan 2014 [29]	799.371 (0.000–2.36 × 10^11^) ^#^	-
Protein			
Animal protein	Jantchou 2010 [17]	2.700 (0.690–10.520) *	-
Vegetable protein	Jantchou 2010 [17]	1.040 (0.280–3.800) *	-

^#^ Based on the data adjusted by smoking and total energy intake; * The highest vs. the lowest category (the mean intake per category was unavailable); SFA, saturated fatty acid; MUFA, monounsaturated fatty acid; PUFA, polyunsaturated fatty acid; DHA, docosahexaenoic acid; EPA, eicosapentaenoic acid.

**Table 3:** Subgroup analyses (random effect model) of carbohydrate, fiber, fat and protein intake (per 10 g increment/day) with the risk of Crohn’s disease.

Subgroup	Carbohydrate		Fibre		Fat		Protein	
	RR (95% CI)	*I*^2^ (%)	RR (95% CI)	*I*^2^ (%)	RR (95% CI)	*I*^2^ (%)	RR (95% CI)	*I*^2^ (%)
Study design								
Case-control	0.991 (0.974–1.008)	19.5	0.815 (0.679–0.980)	0.0	1.026 (0.930–1.132)	70.1	1.008 (0.922–1.101)	57.2
Prospective-cohort	0.997 (0.969–1.026)	-	0.877 (0.761–1.012)	-	1.005 (0.965–1.048)	0.0	1.099 (0.989–1.221)	-
Cohort								
Caucasian	0.999 (0.981–1.018)	0.0	0.844 (0.751–0.947)	0.0	0.997 (0.963–1.033)	0.0	1.015 (0.915–1.126)	63.8
Asian	0.983 (0.965–1.001)	-	1.037 (0.644–1.668)	-	1.134 (1.030–1.249)	-	1.066 (0.981–1.159)	-
Adjusted for smoking								
Yes	0.987 (0.972–1.002)	0.0	0.890 (0.776–1.020)	0.0	1.045 (0.970–1.127)	62.5	1.015 (0.915–1.126)	63.8
No	1.001 (0.975–1.028)	14.4	0.782 (0.641–0.954)	0.0	0.977 (0.916–1.043)	0.0	1.066 (0.981–1.159)	-
